# Acute myocarditis during adjuvant therapies for breast cancer: a case report

**DOI:** 10.1186/s40792-023-01626-7

**Published:** 2023-03-23

**Authors:** Yumiko Ushiyama, Yoshiya Horimoto, Toshitaka Uomori, Yumiko Ishizuka, Misato Okazaki, Hiroko Onagi, Takuo Hayashi, Junichiro Watanabe, Mitsue Saito

**Affiliations:** 1 Department of Breast Oncology, Juntendo University School of Medicine, 2-1-1 Hongo, Bunkyo-Ku, Tokyo, 113-0033 Japan; 2 Department of Human Pathology, Juntendo University School of Medicine, 2-1-1 Hongo, Bunkyo-Ku, Tokyo, 113-0033 Japan

**Keywords:** Acute viral myocarditis, Fulminant myocarditis, Breast cancer, Adjuvant treatment, Hepatic dysfunction

## Abstract

**Background:**

With the improvement of optimal perioperative drug therapy for breast cancer patients, physicians now have to treat the adverse effects and comorbidities associated with long-term treatments. We report a case who suffered cardiac arrest due to acute myocarditis developed after initiation of adjuvant treatment.

**Case presentation:**

After completing preoperative chemotherapy and undergoing curative surgery for right breast cancer, a 46-year-old female patient started adjuvant tamoxifen and resumed trastuzumab. Two months later, she complained fever and dyspnea. Blood tests showed a marked increase in hepatic enzymes, and the patient was rushed to our emergency room on suspicion of drug-induced liver injury. In the emergency room, the patient went into cardiac arrest shortly after tachycardia with ST-segment elevation appeared on the monitored electrocardiogram. Resuscitation was started immediately and tracheal intubation, intra-aortic balloon pumping, and extracorporeal membrane oxygenation were started. Coronary angiography results were negative for ischemic heart disease. A diagnosis of fulminant myocarditis was made and steroid pulse therapy and immunoglobulin therapy were started. After the start of treatment, the symptoms of heart failure improved steadily and the patient was discharged on the 28th day. Histological findings of the myocardial biopsy revealed degeneration and necrosis of myocardial cells with marked lymphocytic infiltration, consistent with the histology of lymphocytic myocarditis. Serum cytomegalovirus, coxsackie B virus and adenovirus antibodies were all elevated and these findings were consistent with acute viral myocarditis.

**Conclusions:**

We report a case with strong indications for therapy-induced liver damage, who was ultimately diagnosed with acute viral myocarditis and successfully treated with multidisciplinary therapy. We believe that our findings would be useful for other clinicians in managing similar patients.

## Background

The outcome for early stage breast cancer (EBC) patients is continually being improved by optimal perioperative drug therapy. This is particularly evident in patients with estrogen receptor (ER)-positive disease, which accounts for the majority of breast cancers [1, 2]. However, physicians are now increasingly having to monitor and treat the adverse effects and comorbidities associated with long-term treatments, such as prolonged (up to 10 years) tamoxifen (TAM) therapy for ER-positive premenopausal EBC patients. Herein, we report the details for a case who suffered cardiac arrest due to acute myocarditis developed after initiation of adjuvant endocrine therapy and resumption of anti-human epidermal growth factor receptor 2 (HER2) therapy.

## Case presentation

A 46-year-old female patient was previously diagnosed with breast cancer (right breast, cT2N1M0 Stage IIB, invasive ductal carcinoma, ER-positive, HER2-positive) at the age of 44. After initially completing preoperative chemotherapy (anthracycline and taxane plus anti-HER2 therapy), the patient underwent curative surgery. The final pathological diagnosis was pathological complete response (ypT0N0). Adjuvant treatment of 10-year TAM concomitantly with 1-year trastuzumab was planned. There was no previous or family history of cardiac disease, and no abnormal findings on cardiac function evaluated after surgery. The patient had a history of hepatitis C and was treated with antiviral agents (sofosbuvir and ribavirin) for 12 weeks resulting in a sustained virological response (SVR).

Two months after starting TAM and resuming trastuzumab, the patient visited her general physician with a complaint of fever and dyspnea. Blood tests showed a marked increase in hepatic enzymes, and the patient was rushed to our emergency room on suspicion of drug-induced liver injury. Upon arrival at the hospital, the patient was conscious, temperature was 36.6 °C, blood pressure was 110/70 mmHg, pulse was 80 bpm, respiratory rate was 20 breaths/min, and SpO_2_ was 99% (in-room air). Blood tests revealed elevated hepatic enzymes, decreased coagulability, thrombocytopenia and marked metabolic acidosis. Table 1A shows the blood test findings at the time of the emergency room visit. The patient was observed in the emergency room with bed rest and intravenous fluids, while the hospitalization process was underway based on the initial diagnosis of drug-induced fulminant hepatitis caused by TAM. During this time, tachycardia (140 beats/min) with ST-segment elevation appeared on the monitored electrocardiogram (ECG), and the ECG indicated anterior wall myocardial ischemia and third-degree atrioventricular block. Shortly thereafter, the patient went into cardiac arrest. Approximately 24 h had passed since the initial visit to her general physician. Resuscitation was started immediately, and because acute coronary syndrome was suspected, the patient was moved to the cardiac angiography room, where tracheal intubation, intra-aortic balloon pumping, and extracorporeal membrane oxygenation (V-A ECMO) were started.

**Table 1:** Blood test findings

(A)		Normal range	(B)		Normal range
White blood cell (/μL)	11,300	(3600–8900)	CK (U/μL)	761	(47–200)
Hemoglobin (g/dl)	9.0	(11.1–15.2)	CK-MB (U/μL)	24	(0–12)
Platelet (/μL)	59,000	(153,000–346,000)	TroponinT (ng/mL)	1010	(0.000–0.100)
ALT (U/μL)	80	(6–43)	proBNP (pg/mL)	7204	(0.0–125.0)
AST (U/μL)	636	(5–37)			
ALP (U/μL)	446	(38–113)			
LDH (U/μL)	801	(124–222)			
γ-GT (U/μL)	57	(0–75)			
T-Bil (mg/dl)	1.37	(0.40–1.20)			
PT (INR)	1.43	(0.90–1.10)			
FDP-Ddimer (μg/dl)	19.3	(0.0–1.0)			
CRP (mg/dl)	2.75	(0.00–0.29)			
BUN (mg/dl)	22	(9–21)			
Cre (mg/dl)	0.69	(0.50–0.80)			
eGFR	72.7				
Na (mmol/dl)	142	(135–145)			
K (mmol/dl)	4.3	(3.5–5.0)			
Cl (mmol/dl)	108	(96–107)			
Ca (mg/dl)	8.7	(8.8–10.6)			

**Fig. 1:**
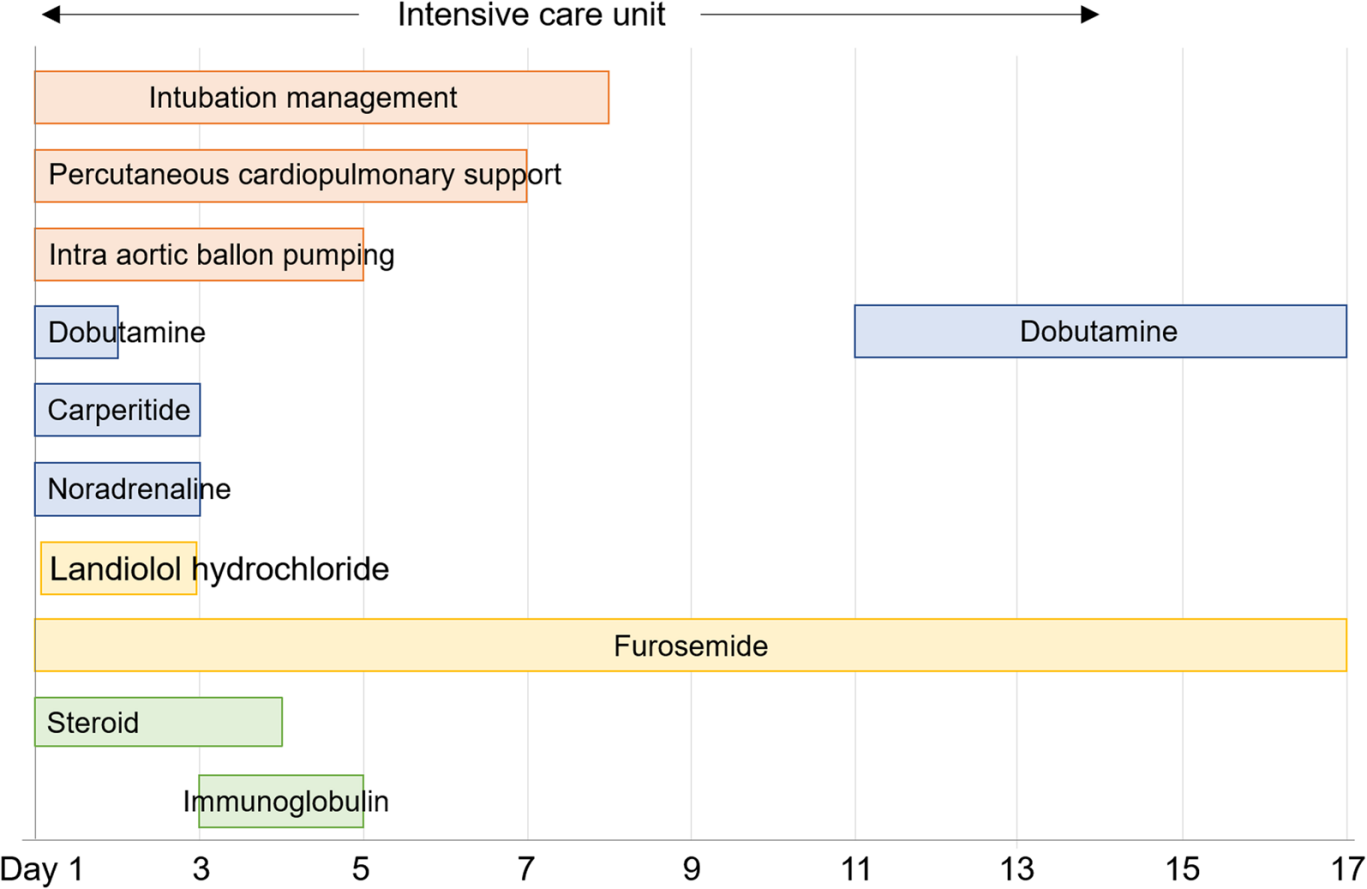
Details of treatments after cardiac arrest. Details of treatments after cardiac arrest are shown

Coronary angiography (CAG) results were negative for ischemic heart disease. A diagnosis of fulminant myocarditis was made based on the pathophysiology and a myocardial biopsy was performed. Blood tests at the time showed CK 761 (U/μL), CK-MB 24 (U/μL), troponin T 1010 (ng/mL) and proBNP 7204 (pg/mL), with a significant increase in myocardial enzymes (Table 1B). Steroid pulse therapy (methylprednisolone 1000 mg × 5 days) and immunoglobulin therapy (1.0 g/day × 2 days) were started immediately after admission. Details of the main treatment after cardiac arrest are shown in Fig. 1. After the start of treatment, the symptoms of heart failure improved steadily and the patient was transferred to a general ward on the 14th day before being discharged on the 28th day.

**Fig. 2:**
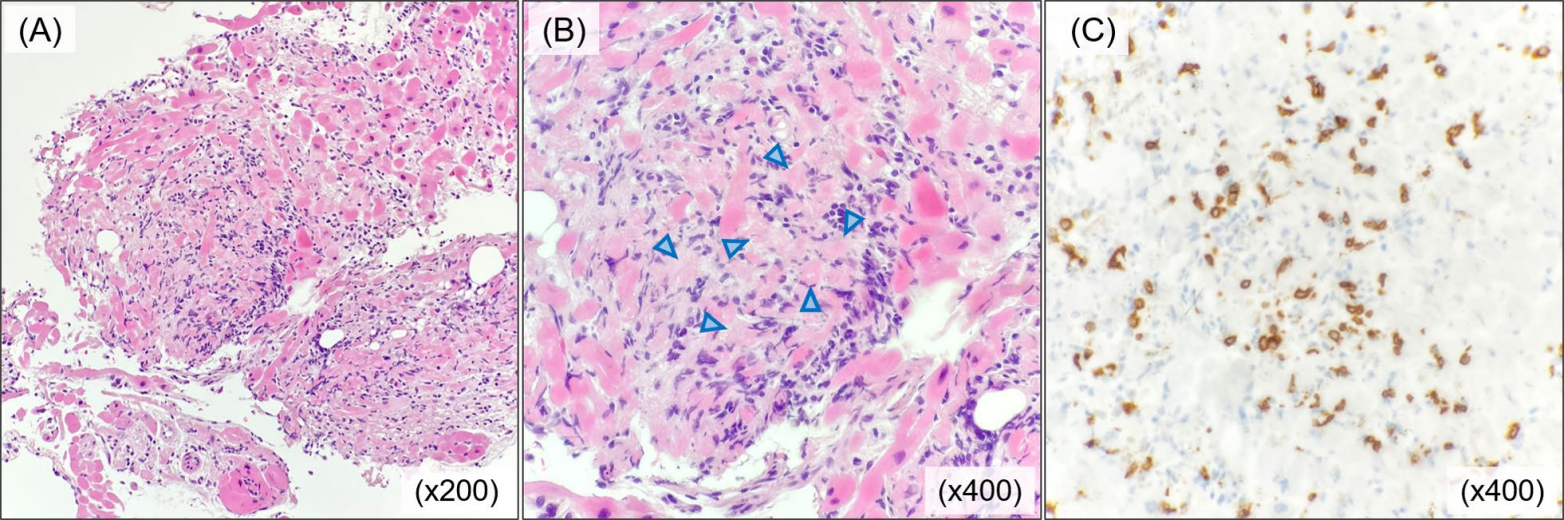
Histological findings of myocardial biopsy. Histological findings of the myocardial biopsy are shown. **A,** **B** Myocardial tissue contains marked infiltration of lymphocytes, histiocytes and cardiomyocytes with degeneration and necrosis (blue arrowheads) evident (hematoxylin and eosin staining). **C** Marked infiltration of CD8-positive T cells was observed

Histological findings of the myocardial biopsy taken at the time of CAG are shown in Fig. 2. Within the myocardial tissue, degeneration and necrosis of myocardial cells were observed with marked lymphocytic infiltration. There was no infiltration of eosinophils or multinucleated giant cells. The infiltrating lymphocytes were predominantly CD8-positive T cells, a finding consistent with the histology of lymphocytic myocarditis. Serum cytomegalovirus, coxsackie B virus and adenovirus antibodies were elevated eightfold, fourfold and fourfold (based on complement fixation test), respectively. Taken together, these findings were consistent with acute viral myocarditis. HCV–RNA was tested before steroid administration and RNA was not detected.

After intensive care, despite the improvement in symptoms of heart failure, the patient’s heart rate remained at 20–30 bpm. Hence, a permanent pacemaker was inserted and the patient was discharged on the 28th day. After discharge from the hospital, an attempt was made to remove the pacemaker, but bradyarrhythmia remained, thus the pacemaker was retained and remains to date. Ten months have passed, since the TAM was recommenced, and the patient remains on a good course with no elevation of liver enzymes or any findings that suggest recurrence of breast cancer.

## Discussion

Since this patient developed hepatic dysfunction relatively early after the administration of TAM, it was initially assumed that the patient had developed drug-induced fulminant hepatitis. However, it became evident this severe liver dysfunction was caused by blood congestion due to myocarditis.

Drug-induced, congestive and viral hepatitis relapse are the most common causes of liver injury; however, it is difficult to make a differential diagnosis between the former two based on clinical findings. It has been reported that in the case of shock liver, both ALT and AST are often elevated, reflecting hepatocyte necrosis in the central venous zone due to ischemia [3], and ALT/LDH and AST/LDH ratios are often both smaller than 1.0 [4]. Retrospectively, the present case fit these criteria, but no specific laboratory markers have been established to differentiate between the dug-induced and congestive hepatitis. In this presented case, there were no signs of circulatory failure at the time of the emergency department visit, making a diagnosis of shock liver due to blood congestion caused by myocarditis challenging. With the recent increase in the use of immune checkpoint inhibitors (ICIs) in the pharmacotherapy of breast cancer, the need to differentiate autoimmune hepatitis is likely to increase in the future and requires physicians’ caution.

Symptoms of acute myocarditis include common cold-like symptoms, gastrointestinal symptoms, such as nausea and vomiting, and signs of heart failure, such as dyspnea. Viral causes are the most common, followed by autoimmune and drug-related causes [5]. In recent years, myocarditis caused by ICI has also been on the increase and requires further attention. Hepatitis C virus is also known to be cause of myocarditis [6]. However, as reactivation after SVR is considered very unlikely [7], the possibility that HCV was the cause was excluded in this case. Pathologically, the disease is divided into lymphocytic, eosinophilic, granulomatous, and giant cell types, with viral myocarditis being classified as lymphocytic. While the overall mortality rate of myocarditis is about 5%, the acute mortality rate is as high as 42% in the fulminant form with circulatory failure, as observed in the present case [8]. Although this case had a rapid course, early intervention by a cardiologist allowed appropriate diagnosis and treatment, and the patient was successfully saved.

## Conclusions

We report a case with strong indications for therapy-induced liver damage, after starting TAM and resumption of trastuzumab, who was ultimately diagnosed with acute viral myocarditis. This patient was successfully treated with multidisciplinary therapy. During drug treatment, it is necessary to take a broad view and differentiate between various symptoms from the preconceived notion that they are side effects of drugs.

## Data Availability

Not applicable.

## Abbreviations

CAG: Coronary angiography
EBC: Early stage breast cancer
ECG: Electrocardiogram
V-A ECMO: Extracorporeal membrane oxygenation
ER: Estrogen receptor
HER2: Human epidermal growth factor receptor 2
ICIs: Immune checkpoint inhibitors
SVR: Sustained virological response
TAM: Tamoxifen
